# 11α,15α-Dihy­droxy­androst-4-ene-3,17-dione

**DOI:** 10.1107/S1600536811038608

**Published:** 2011-09-30

**Authors:** Yan-Bing Shen, Min Wang, Qi-Kun Liang, Jian-Mei Luo

**Affiliations:** a College of Biotechnology, Tianjin University of Science and Technology, Tianjin, 300457 People’s Republic of China

## Abstract

The title compound, C_19_H_26_O_4_, was biotransformed from androstenedione. In the crystal, inter­molecular O—H⋯O hydrogen bonds link molecules into a corrugated sheet, which lies parallel to the *ab* plane. Ring *A* has a slightly distorted half-chair conformation, rings *B* and *C* adopt chair conformations, while the cyclo­pentane ring *D* adopts a 14α-envelope conformation.

## Related literature

For related structures, see: Galdecki *et al.* (1990 ▶); Thamotharan *et al.* (2004 ▶); Vasuki *et al.* (2002 ▶). For details of biotransformation, see: Ahmad *et al.* (1992 ▶); Kollerov *et al.* (2008 ▶); Malaviya & Gomes (2008 ▶); Perez *et al.* (2006 ▶). For conformational analysis, see Cremer & Pople (1975 ▶).
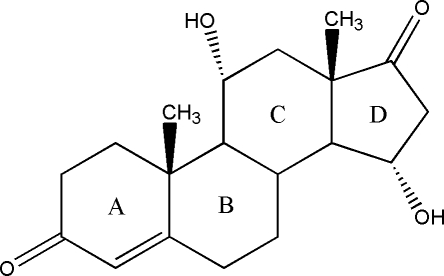


## Experimental

### 

#### Crystal data

C_19_H_26_O_4_
*M* *_r_* = 318.40
Orthorhombic, 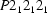

*a* = 7.8716 (8) Å
*b* = 12.2725 (12) Å
*c* = 17.2100 (16) Å
*V* = 1662.6 (3) Å^3^
*Z* = 4
Mo *K*α radiation
μ = 0.09 mm^−1^
*T* = 113 K
0.22 × 0.18 × 0.12 mm

#### Data collection

Rigaku Saturn 724CCD diffractometer
Absorption correction: multi-scan (*CrystalClear*; Rigaku, 2005 ▶) *T*_min_ = 0.981, *T*_max_ = 0.990
17662 measured reflections
2275 independent reflections
2050 reflections with *I* > 2σ(*I*)
*R*_int_ = 0.047

#### Refinement

*R*[*F* ^2^ > 2σ(*F* ^2^)] = 0.037
*wR*(*F* ^2^) = 0.081
*S* = 1.03
2275 reflections
218 parameters
H atoms treated by a mixture of independent and constrained refinement
Δρ_max_ = 0.16 e Å^−3^
Δρ_min_ = −0.20 e Å^−3^

### 

Data collection: *CrystalClear* (Rigaku, 2005 ▶); cell refinement: *CrystalClear*; data reduction: *CrystalClear*; program(s) used to solve structure: *SHELXS97* (Sheldrick, 2008 ▶); program(s) used to refine structure: *SHELXL97* (Sheldrick, 2008 ▶); molecular graphics: *SHELXTL* (Sheldrick, 2008 ▶); software used to prepare material for publication: *SHELXTL*.

## Supplementary Material

Crystal structure: contains datablock(s) global, I. DOI: 10.1107/S1600536811038608/lw2070sup1.cif

Structure factors: contains datablock(s) I. DOI: 10.1107/S1600536811038608/lw2070Isup2.hkl

Additional supplementary materials:  crystallographic information; 3D view; checkCIF report

## Figures and Tables

**Table 1:** Hydrogen-bond geometry (Å, °)

*D*—H⋯*A*	*D*—H	H⋯*A*	*D*⋯*A*	*D*—H⋯*A*
O3—H3⋯O2^i^	0.88 (3)	1.94 (3)	2.800 (2)	164 (3)
O2—H2⋯O1^ii^	0.81 (3)	1.95 (3)	2.7600 (19)	180 (3)

Symmetry codes: (i) 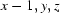; (ii) 
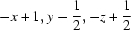
.

**Fig. 1.**
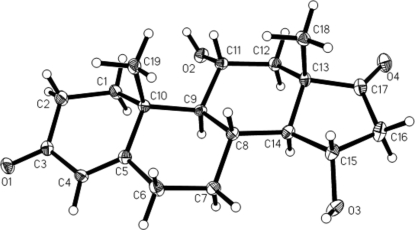
A view of (I) with our numbering scheme. Displacement ellipsoids are drawn at the 30% probability level.

**Fig. 2.**
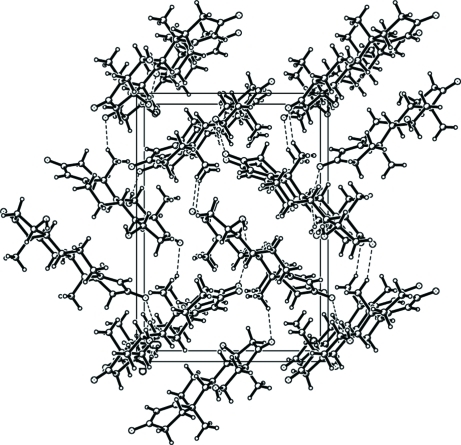
View of the packing of the title compound

## supplementary crystallographic information

### Comment

Androst-4-ene-3,17-dione (AD) is the important intermediate in the pharmaceutical industry (Perez *et al.*, 2006; Kollerov *et al.* 2008). The production of several high value steroid drugs is mostly derived from key compounds such as AD by chemical synthesis (Ahmad *et al.*, 1992; Malaviya & Gomes, 2008).

The structure of the title compound is depicted in Fig. 1. The 11α,15α-dihydroxy-androstenedione has three six-membered rings (A/B/C) and one five-membered rings (D). Ring A has a slightly distorted half-chair conformation. Rings B and C adopt chair conformations, while the cyclopentane ring D adopts a 14α-envelope conformation. The torsion angle C8—C9—C11—O2 = 162.83 (13), indicates that the 11-hydroxy has an α configuration. The 15-hydroxy has an α configuration with the torsion angle C13—C14—C15—O3 = -160.83 (14)°. The bond lengths and angles are within normal ranges (Thamotharan *et al.*, 2004; Vasuki, *et al.*, 2002; Galdecki *et al.*, 1990).

Two types of intermolecular hydrogen bonds contribute to the formation of a two-dimensional corrugated sheet lying parallel to the *ab*-plane, Figure 2. The O3 hydroxyl hydrogen forms a hydrogen bond to hydroxyl atom O2 at (1-x,y,z) by unit translation along the a-axis. Hydroxyl oxygen O2 forms a hydrogen bond to the screw-related carbonyl atom O1 at (1-x,-1/2+y,1/2-z), Table 1.

### Experimental

Experimental

Reagents: *Colletotrichum lini* AS3. 4486 was obtained from Institute of Microbiology, Chinese Academy of Sciences and maintained on Potato Dextrose Agar at 4°C. Androst-4-en-3,17-dione was obtained from Tianjin Pharmaceutical Company.

Cultures Protocol: Colletotrichum lini AS3. 4486 was cultivated in shake flasks in two consecutive cultivation steps: 72 h for seed culture and 24 h for cell cultivation. Seed medium comprised glucose 30 g/L, corn steep liquor 10 g/L and tap water (pH 7.0). Cell cultivation medium comprised glucose 3 g/L, corn steep liquor 10 g/L, soy meal 10 g/L, NaNO_3_ 2 g/L, KH_2_PO_4_, 1 g/L, K_2_HPO_4_, 2 g/L, MgSO_4_.7H_2_O 0.5g/L, KCl, 0.5g/L, FeSO_4_.7H_2_O, 0.02 g/L, (pH 7.0). Cells were grown in 250 ml shake flasks containing 50 ml culture medium on a rotary shaker (200 r/min) at 25°C using 10% (v/v) of the seed culture as inoculum.

Biotransformation: 50 mg of the androst-4-en-3,17-dione dissolved in 1 ml of ethanol was added to the culture after 24 h for growth and the reaction was allowed to proceed for 72 h. The mycelium was then removed by filtration.

Separation and purification: The biomass and the broth were extracted separately with EtOAc. All extracts were combined and dried (anhydr. MgSO_4_). The solvents after filtration were evaporated under reduced pressure. The crude extracts were purified by Si gel column using dichloromethane/ether/methanol (25:2:1, v/v/v). The white powder was diffused with n-hexane/acetone at room temperature. Colorless prismatic crystals suitable for X-ray analysis were obtained.

### Refinement

In the absence of significant anomalous dispersion effects, Freidel pairs were merged. All H atoms of O—H were initially located in a difference Fourier map and were refined with the restraints O—H bond lengths ranging 0.81 (3)–0.88 (3). O—H = 0.81 - 0.99 Å. Other H atoms were positioned geometrically and refined using a riding model, with d(C—H) = 0.95 - 1.00Å and *U*_iso_(H) = 1.2*U*_eq_(C) or 1.5*U*_eq_(C_methyl_). The absolute configuration was assumed since the structure of the commercially obtained androst-4-en-3,17-dione used in the preparation was known.

### Crystal data

**Table:**

C_19_H_26_O_4_	*D*_x_ = 1.272 Mg m^−^^3^
*M**_r_* = 318.40	Mo *K*α radiation, λ = 0.71073 Å
Orthorhombic, *P*2_1_2_1_2_1_	Cell parameters from 6101 reflections
*a* = 7.8716 (8) Å	θ = 2.0–28.0°
*b* = 12.2725 (12) Å	µ = 0.09 mm^−^^1^
*c* = 17.2100 (16) Å	*T* = 113 K
*V* = 1662.6 (3) Å^3^	Prism, colourless
*Z* = 4	0.22 × 0.18 × 0.12 mm
*F*(000) = 688	

### Data collection

**Table:**

Rigaku Saturn 724CCD diffractometer	2275 independent reflections
Radiation source: rotating anode	2050 reflections with *I* > 2σ(*I*)
multilayer	*R*_int_ = 0.047
Detector resolution: 14.22 pixels mm^-1^	θ_max_ = 27.9°, θ_min_ = 2.0°
ω scans	*h* = −10→10
Absorption correction: multi-scan (*CrystalClear*; Rigaku, 2005)	*k* = −14→16
*T*_min_ = 0.981, *T*_max_ = 0.990	*l* = −21→22
17662 measured reflections	

### Refinement

**Table:**

Refinement on *F*^2^	Primary atom site location: structure-invariant direct methods
Least-squares matrix: full	Secondary atom site location: difference Fourier map
*R*[*F*^2^ > 2σ(*F*^2^)] = 0.037	Hydrogen site location: inferred from neighbouring sites
*wR*(*F*^2^) = 0.081	H atoms treated by a mixture of independent and constrained refinement
*S* = 1.03	*w* = 1/[σ^2^(*F*_o_^2^) + (0.0478*P*)^2^] where *P* = (*F*_o_^2^ + 2*F*_c_^2^)/3
2275 reflections	(Δ/σ)_max_ < 0.001
218 parameters	Δρ_max_ = 0.16 e Å^−^^3^
0 restraints	Δρ_min_ = −0.20 e Å^−^^3^

### Special details

**Table:**

Experimental. Rigaku *CrystalClear*-SM Expert 2.0 r2
Geometry. All e.s.d.'s (except the e.s.d. in the dihedral angle between two l.s. planes) are estimated using the full covariance matrix. The cell e.s.d.'s are taken into account individually in the estimation of e.s.d.'s in distances, angles and torsion angles; correlations between e.s.d.'s in cell parameters are only used when they are defined by crystal symmetry. An approximate (isotropic) treatment of cell e.s.d.'s is used for estimating e.s.d.'s involving l.s. planes.
Refinement. Refinement of *F*^2^ against ALL reflections. The weighted *R*-factor *wR* and goodness of fit *S* are based on *F*^2^, conventional *R*-factors *R* are based on *F*, with *F* set to zero for negative *F*^2^. The threshold expression of *F*^2^ > σ(*F*^2^) is used only for calculating *R*-factors(gt) *etc*. and is not relevant to the choice of reflections for refinement. *R*-factors based on *F*^2^ are statistically about twice as large as those based on *F*, and *R*- factors based on ALL data will be even larger.

### Fractional atomic coordinates and isotropic or equivalent isotropic displacement parameters (Å^2^)

**Table:**

	*x*	*y*	*z*	*U*_iso_*/*U*_eq_	
O1	0.31267 (17)	0.53408 (9)	0.26929 (8)	0.0327 (3)	
O2	0.51158 (16)	0.08691 (11)	0.09715 (8)	0.0284 (3)	
H2	0.563 (3)	0.0711 (19)	0.1363 (15)	0.056 (8)*	
O3	−0.27423 (18)	0.05176 (12)	−0.03028 (8)	0.0328 (3)	
H3	−0.357 (4)	0.064 (2)	0.0029 (16)	0.071 (9)*	
O4	0.16087 (19)	−0.21111 (10)	−0.06034 (7)	0.0349 (4)	
C1	0.4080 (2)	0.26310 (14)	0.19535 (12)	0.0286 (4)	
H1A	0.4888	0.2042	0.2087	0.034*	
H1B	0.4428	0.2935	0.1445	0.034*	
C2	0.4205 (2)	0.35273 (15)	0.25684 (12)	0.0309 (4)	
H2A	0.4037	0.3203	0.3089	0.037*	
H2B	0.5357	0.3850	0.2553	0.037*	
C3	0.2921 (2)	0.44021 (14)	0.24442 (10)	0.0238 (4)	
C4	0.1357 (2)	0.40863 (14)	0.20623 (10)	0.0232 (4)	
H4	0.0535	0.4636	0.1964	0.028*	
C5	0.1002 (2)	0.30692 (13)	0.18408 (10)	0.0214 (4)	
C6	−0.0777 (2)	0.28022 (15)	0.15829 (11)	0.0299 (4)	
H6A	−0.1398	0.3489	0.1482	0.036*	
H6B	−0.1371	0.2415	0.2008	0.036*	
C7	−0.0825 (2)	0.21006 (14)	0.08549 (11)	0.0267 (4)	
H7A	−0.0385	0.2522	0.0408	0.032*	
H7B	−0.2012	0.1890	0.0740	0.032*	
C8	0.0253 (2)	0.10785 (13)	0.09697 (10)	0.0202 (4)	
H8	−0.0158	0.0696	0.1447	0.024*	
C9	0.2132 (2)	0.14340 (14)	0.11087 (9)	0.0185 (3)	
H9	0.2434	0.1930	0.0669	0.022*	
C10	0.2289 (2)	0.21395 (13)	0.18739 (10)	0.0205 (4)	
C11	0.3406 (2)	0.04730 (14)	0.10728 (10)	0.0213 (4)	
H11	0.3346	0.0064	0.1575	0.026*	
C12	0.3116 (2)	−0.03257 (14)	0.04087 (10)	0.0236 (4)	
H12A	0.3401	0.0030	−0.0091	0.028*	
H12B	0.3875	−0.0963	0.0474	0.028*	
C13	0.1278 (2)	−0.07058 (13)	0.03913 (9)	0.0212 (4)	
C14	0.0122 (2)	0.02890 (13)	0.02873 (9)	0.0216 (4)	
H14	0.0545	0.0688	−0.0181	0.026*	
C15	−0.1598 (2)	−0.02117 (14)	0.00633 (10)	0.0257 (4)	
H15	−0.2147	−0.0537	0.0533	0.031*	
C16	−0.1069 (3)	−0.11229 (16)	−0.05010 (11)	0.0328 (5)	
H16A	−0.1820	−0.1765	−0.0440	0.039*	
H16B	−0.1139	−0.0866	−0.1045	0.039*	
C17	0.0746 (2)	−0.14129 (15)	−0.02929 (10)	0.0262 (4)	
C18	0.0840 (2)	−0.13973 (14)	0.11174 (9)	0.0256 (4)	
H18A	−0.0316	−0.1685	0.1067	0.038*	
H18B	0.0913	−0.0940	0.1583	0.038*	
H18C	0.1645	−0.2003	0.1161	0.038*	
C19	0.1869 (3)	0.14572 (15)	0.26033 (10)	0.0325 (5)	
H19A	0.2029	0.1905	0.3069	0.049*	
H19B	0.2625	0.0824	0.2627	0.049*	
H19C	0.0686	0.1210	0.2577	0.049*	

### Atomic displacement parameters (Å^2^)

**Table:**

	*U*^11^	*U*^22^	*U*^33^	*U*^12^	*U*^13^	*U*^23^
O1	0.0360 (8)	0.0215 (6)	0.0407 (8)	−0.0023 (6)	−0.0052 (6)	−0.0074 (6)
O2	0.0162 (6)	0.0369 (7)	0.0322 (7)	0.0014 (6)	−0.0021 (6)	−0.0035 (6)
O3	0.0253 (7)	0.0453 (8)	0.0277 (7)	0.0002 (7)	−0.0082 (6)	0.0004 (6)
O4	0.0415 (9)	0.0337 (7)	0.0296 (7)	−0.0005 (7)	0.0077 (6)	−0.0113 (6)
C1	0.0193 (9)	0.0242 (10)	0.0422 (11)	0.0024 (8)	−0.0062 (8)	−0.0097 (8)
C2	0.0246 (9)	0.0266 (9)	0.0414 (11)	−0.0001 (8)	−0.0094 (9)	−0.0079 (9)
C3	0.0274 (9)	0.0224 (8)	0.0217 (8)	−0.0033 (8)	0.0029 (7)	−0.0018 (7)
C4	0.0235 (9)	0.0214 (9)	0.0246 (8)	0.0046 (7)	0.0005 (7)	−0.0006 (7)
C5	0.0210 (9)	0.0227 (9)	0.0206 (8)	−0.0002 (7)	0.0018 (7)	−0.0007 (7)
C6	0.0181 (9)	0.0265 (9)	0.0452 (11)	0.0025 (8)	−0.0018 (8)	−0.0105 (9)
C7	0.0187 (9)	0.0256 (9)	0.0357 (10)	0.0004 (8)	−0.0058 (8)	−0.0030 (8)
C8	0.0158 (8)	0.0217 (8)	0.0233 (8)	−0.0014 (7)	−0.0008 (7)	−0.0004 (7)
C9	0.0162 (8)	0.0193 (8)	0.0201 (8)	0.0001 (7)	−0.0008 (6)	0.0020 (7)
C10	0.0206 (9)	0.0189 (8)	0.0221 (8)	−0.0005 (7)	−0.0024 (7)	−0.0004 (7)
C11	0.0169 (8)	0.0233 (8)	0.0238 (8)	0.0007 (7)	−0.0006 (7)	0.0007 (7)
C12	0.0219 (9)	0.0248 (9)	0.0243 (8)	0.0023 (8)	0.0022 (7)	−0.0041 (7)
C13	0.0233 (9)	0.0219 (9)	0.0186 (8)	−0.0019 (7)	0.0027 (7)	−0.0048 (7)
C14	0.0184 (8)	0.0253 (9)	0.0212 (8)	−0.0024 (7)	0.0005 (7)	0.0001 (7)
C15	0.0223 (9)	0.0315 (10)	0.0234 (9)	−0.0038 (8)	−0.0026 (7)	−0.0024 (8)
C16	0.0302 (10)	0.0405 (11)	0.0275 (9)	−0.0075 (9)	0.0003 (8)	−0.0099 (9)
C17	0.0311 (10)	0.0269 (9)	0.0206 (8)	−0.0068 (9)	0.0063 (8)	−0.0026 (8)
C18	0.0323 (10)	0.0219 (8)	0.0225 (8)	0.0027 (8)	0.0041 (8)	−0.0014 (7)
C19	0.0553 (13)	0.0225 (8)	0.0197 (8)	−0.0005 (10)	−0.0029 (9)	−0.0007 (8)

### Geometric parameters (Å, °)

**Table:**

O1—C3	1.240 (2)	C8—H8	1.0000
O2—C11	1.442 (2)	C9—C11	1.549 (2)
O2—H2	0.81 (3)	C9—C10	1.581 (2)
O3—C15	1.417 (2)	C9—H9	1.0000
O3—H3	0.88 (3)	C10—C19	1.545 (2)
O4—C17	1.217 (2)	C11—C12	1.523 (2)
C1—C2	1.530 (2)	C11—H11	1.0000
C1—C10	1.539 (2)	C12—C13	1.521 (2)
C1—H1A	0.9900	C12—H12A	0.9900
C1—H1B	0.9900	C12—H12B	0.9900
C2—C3	1.490 (2)	C13—C17	1.521 (2)
C2—H2A	0.9900	C13—C14	1.533 (2)
C2—H2B	0.9900	C13—C18	1.549 (2)
C3—C4	1.449 (2)	C14—C15	1.536 (2)
C4—C5	1.335 (2)	C14—H14	1.0000
C4—H4	0.9500	C15—C16	1.539 (2)
C5—C6	1.505 (2)	C15—H15	1.0000
C5—C10	1.527 (2)	C16—C17	1.515 (3)
C6—C7	1.521 (2)	C16—H16A	0.9900
C6—H6A	0.9900	C16—H16B	0.9900
C6—H6B	0.9900	C18—H18A	0.9800
C7—C8	1.527 (2)	C18—H18B	0.9800
C7—H7A	0.9900	C18—H18C	0.9800
C7—H7B	0.9900	C19—H19A	0.9800
C8—C14	1.526 (2)	C19—H19B	0.9800
C8—C9	1.561 (2)	C19—H19C	0.9800
			
C11—O2—H2	106.5 (18)	C19—C10—C9	111.30 (12)
C15—O3—H3	107.0 (18)	O2—C11—C12	105.43 (13)
C2—C1—C10	113.73 (15)	O2—C11—C9	110.63 (13)
C2—C1—H1A	108.8	C12—C11—C9	115.02 (13)
C10—C1—H1A	108.8	O2—C11—H11	108.5
C2—C1—H1B	108.8	C12—C11—H11	108.5
C10—C1—H1B	108.8	C9—C11—H11	108.5
H1A—C1—H1B	107.7	C13—C12—C11	110.77 (14)
C3—C2—C1	112.03 (15)	C13—C12—H12A	109.5
C3—C2—H2A	109.2	C11—C12—H12A	109.5
C1—C2—H2A	109.2	C13—C12—H12B	109.5
C3—C2—H2B	109.2	C11—C12—H12B	109.5
C1—C2—H2B	109.2	H12A—C12—H12B	108.1
H2A—C2—H2B	107.9	C12—C13—C17	116.88 (14)
O1—C3—C4	121.08 (17)	C12—C13—C14	108.82 (13)
O1—C3—C2	122.11 (16)	C17—C13—C14	101.58 (13)
C4—C3—C2	116.67 (14)	C12—C13—C18	111.33 (15)
C5—C4—C3	123.90 (17)	C17—C13—C18	104.51 (13)
C5—C4—H4	118.0	C14—C13—C18	113.48 (14)
C3—C4—H4	118.0	C8—C14—C13	112.07 (13)
C4—C5—C6	118.85 (16)	C8—C14—C15	120.43 (14)
C4—C5—C10	123.31 (16)	C13—C14—C15	103.52 (13)
C6—C5—C10	117.76 (14)	C8—C14—H14	106.7
C5—C6—C7	112.89 (15)	C13—C14—H14	106.7
C5—C6—H6A	109.0	C15—C14—H14	106.7
C7—C6—H6A	109.0	O3—C15—C14	114.78 (14)
C5—C6—H6B	109.0	O3—C15—C16	110.50 (15)
C7—C6—H6B	109.0	C14—C15—C16	102.16 (15)
H6A—C6—H6B	107.8	O3—C15—H15	109.7
C6—C7—C8	110.18 (14)	C14—C15—H15	109.7
C6—C7—H7A	109.6	C16—C15—H15	109.7
C8—C7—H7A	109.6	C17—C16—C15	106.06 (15)
C6—C7—H7B	109.6	C17—C16—H16A	110.5
C8—C7—H7B	109.6	C15—C16—H16A	110.5
H7A—C7—H7B	108.1	C17—C16—H16B	110.5
C14—C8—C7	112.61 (13)	C15—C16—H16B	110.5
C14—C8—C9	111.06 (13)	H16A—C16—H16B	108.7
C7—C8—C9	108.46 (13)	O4—C17—C16	126.00 (17)
C14—C8—H8	108.2	O4—C17—C13	126.01 (18)
C7—C8—H8	108.2	C16—C17—C13	107.96 (15)
C9—C8—H8	108.2	C13—C18—H18A	109.5
C11—C9—C8	113.24 (13)	C13—C18—H18B	109.5
C11—C9—C10	113.54 (12)	H18A—C18—H18B	109.5
C8—C9—C10	110.80 (13)	C13—C18—H18C	109.5
C11—C9—H9	106.2	H18A—C18—H18C	109.5
C8—C9—H9	106.2	H18B—C18—H18C	109.5
C10—C9—H9	106.2	C10—C19—H19A	109.5
C5—C10—C1	108.55 (13)	C10—C19—H19B	109.5
C5—C10—C19	107.05 (14)	H19A—C19—H19B	109.5
C1—C10—C19	109.65 (15)	C10—C19—H19C	109.5
C5—C10—C9	109.03 (13)	H19A—C19—H19C	109.5
C1—C10—C9	111.13 (14)	H19B—C19—H19C	109.5
			
C10—C1—C2—C3	−53.7 (2)	C10—C9—C11—O2	−69.69 (17)
C1—C2—C3—O1	−155.82 (17)	C8—C9—C11—C12	43.52 (19)
C1—C2—C3—C4	28.3 (2)	C10—C9—C11—C12	171.00 (14)
O1—C3—C4—C5	−174.15 (17)	O2—C11—C12—C13	−173.29 (13)
C2—C3—C4—C5	1.8 (3)	C9—C11—C12—C13	−51.13 (19)
C3—C4—C5—C6	169.05 (16)	C11—C12—C13—C17	173.62 (14)
C3—C4—C5—C10	−7.7 (3)	C11—C12—C13—C14	59.39 (18)
C4—C5—C6—C7	136.19 (17)	C11—C12—C13—C18	−66.41 (17)
C10—C5—C6—C7	−46.9 (2)	C7—C8—C14—C13	177.14 (14)
C5—C6—C7—C8	53.8 (2)	C9—C8—C14—C13	55.28 (18)
C6—C7—C8—C14	174.60 (14)	C7—C8—C14—C15	−60.8 (2)
C6—C7—C8—C9	−62.07 (19)	C9—C8—C14—C15	177.33 (15)
C14—C8—C9—C11	−44.45 (18)	C12—C13—C14—C8	−63.21 (17)
C7—C8—C9—C11	−168.71 (14)	C17—C13—C14—C8	172.92 (14)
C14—C8—C9—C10	−173.35 (13)	C18—C13—C14—C8	61.33 (18)
C7—C8—C9—C10	62.39 (17)	C12—C13—C14—C15	165.52 (14)
C4—C5—C10—C1	−16.8 (2)	C17—C13—C14—C15	41.65 (16)
C6—C5—C10—C1	166.38 (16)	C18—C13—C14—C15	−69.93 (17)
C4—C5—C10—C19	101.45 (19)	C8—C14—C15—O3	73.1 (2)
C6—C5—C10—C19	−75.32 (18)	C13—C14—C15—O3	−160.83 (14)
C4—C5—C10—C9	−138.03 (16)	C8—C14—C15—C16	−167.34 (15)
C6—C5—C10—C9	45.2 (2)	C13—C14—C15—C16	−41.22 (17)
C2—C1—C10—C5	46.6 (2)	O3—C15—C16—C17	147.08 (15)
C2—C1—C10—C19	−70.07 (19)	C14—C15—C16—C17	24.51 (18)
C2—C1—C10—C9	166.46 (14)	C15—C16—C17—O4	179.09 (17)
C11—C9—C10—C5	179.05 (13)	C15—C16—C17—C13	1.08 (18)
C8—C9—C10—C5	−52.21 (17)	C12—C13—C17—O4	37.5 (2)
C11—C9—C10—C1	59.44 (18)	C14—C13—C17—O4	155.76 (17)
C8—C9—C10—C1	−171.82 (14)	C18—C13—C17—O4	−86.0 (2)
C11—C9—C10—C19	−63.08 (18)	C12—C13—C17—C16	−144.46 (16)
C8—C9—C10—C19	65.66 (18)	C14—C13—C17—C16	−26.23 (17)
C8—C9—C11—O2	162.83 (13)	C18—C13—C17—C16	92.00 (16)

### Hydrogen-bond geometry (Å, °)

**Table:**

*D*—H···*A*	*D*—H	H···*A*	*D*···*A*	*D*—H···*A*
O3—H3···O2^i^	0.88 (3)	1.94 (3)	2.800 (2)	164 (3)
O2—H2···O1^ii^	0.81 (3)	1.95 (3)	2.7600 (19)	180 (3)

Symmetry codes: (i) *x*−1, *y*, *z*; (ii) −*x*+1, *y*−1/2, −*z*+1/2.
